# PULMONARY MUCORMYCOSIS MIMICKING AS PULMONARY TUBERCULOSIS: A CASE REPORT

**DOI:** 10.4103/0970-2113.59595

**Published:** 2008

**Authors:** Rajiv Garg, Rungmei SK Marak, Sanjay Kumar Verma, Jagdeep Singh, Sanjay, Rajendra Prasad

**Affiliations:** 1 Assistant Professor Department of Pulmonary Medicine, Sanjay Gandhi Post Graduate Institute of Medical Sciences, Lucknow; 3 Senior Resident Department of Pulmonary Medicine, Sanjay Gandhi Post Graduate Institute of Medical Sciences, Lucknow; 5 Junior Resident Department of Pulmonary Medicine, Sanjay Gandhi Post Graduate Institute of Medical Sciences, Lucknow; 6 Professor & Head Department of Pulmonary Medicine, Sanjay Gandhi Post Graduate Institute of Medical Sciences, Lucknow; 2 Assistant Professor Dept. of Microbiology, Sanjay Gandhi Post Graduate Institute of Medical Sciences, Lucknow; 4 Senior Resident Dept. of Microbiology, Sanjay Gandhi Post Graduate Institute of Medical Sciences, Lucknow

**Keywords:** Pulmonary, Mucormycosis, Tuberculosis

## Abstract

Pulmonary Mucormycosis is an uncommon disease caused by fungi of class Zygomycetes. It occurs predminantly in an immunodeficient host most common risk factor being diabetes mellitus. The lesions are localized in the lungs or the mediastinum. We are reporting a case of 70 years old male, having cough, haemoptysis, fever and chest pain. He was on antituberculosis treatment (RHEZ) for last 10 days and was later found to have Pulmonary Mucormycosis on further evaluation.

## INTRODUCTION

Pulmonary Mucormycosis is an uncommon disease caused by fungi of class Zygomycetes, affecting immunocompromised hosts, both in developing and developed countries.^1^ It is the second most common form of mucormycosis, next only to Rhinocerebral disease and accounts for more than 30 % of the disease.^2^ Its clinical presentation in lung is defined as acute, if symptoms are present for less than 30 days. We are reporting a case of an acute pulmonary mucormycosis associated with diabetes mellitus. It was diagnosed unusually by trans-thoracic fine needle aspirate examination.

## CASE REPORT

**Fig 1:**
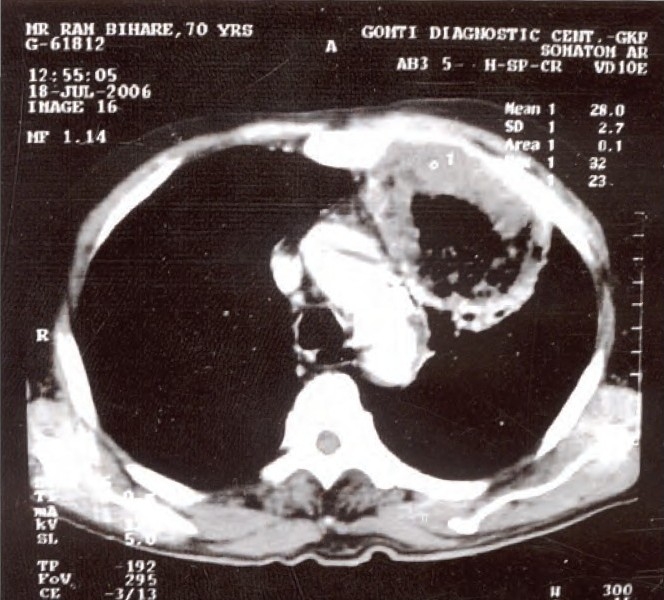
Computed tomography revealed large thick walled cavity on left side in left upper lobe abutting chest wall and encroaching towards arch of aorta.

A 70 year-old male, smoker for 50 years, presented with history of fever, cough, chest pain, mucopurulent expectoration and recurrent haemoptysis for last 25 days. He was given symptomatic treatment for haemoptysis. Patient was also taking antituberculosis treatment (Rifampicin, Isoniazid, Ethambutol, Pyrazinamide) for last 10 days after which he developed drug induced hepatitis. So, he was referred to us. On General examination, patient was emaciated. His pulse rate was 94/min, blood pressure, 120/70 mmHg and respiratory rate was 28/min. Examination of the respiratory system revealed bronchial breath sound over left mammary area. Chest radiograph revealed presence of air space consolidation in mid zone of the left lung. Computed tomography revealed large thick walled cavity on left side in left upper lobe abutting chest wall and encroaching towards arch of aorta (Fig. 1).

Serial investigations showed uncontrolled blood sugar in the range of 232-360 mg/dL. A complete blood count showed hemoglobin of 11.4 gm/dL, total leukocyte count of 7500 cells/cmm and differential count of 70% neutrophils, 29% lymphocytes and 1% eosinophils. Sputum examination did not reveal acid fast bacilli (AFB) on Ziehl-Neelson staining and sputum culture for pyogenic organisms was also sterile after 48 hours of incubation. Mantoux test (10 tuberculin units) showed indurations of 6mm at 72 hours.

**Fig 2:**
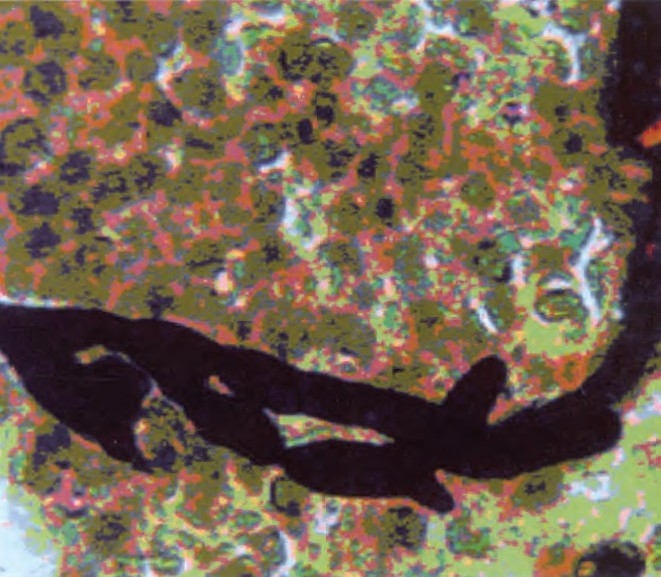
GMS (Gomori's Methanamine Silver staining) from Fine Needle Aspiration sample showing broad randomly branched and aseptate hyphae of Mucormycosis.

He was put on antibiotics (Co-amoxiclav and clindamycin) for 2 weeks without any clinical and radiological response. Patient refused for bronchoscopy. Transthoracic needle aspiration from left cavitary lesion was done, and sent for AFB smear, malignant cells and fungal smear examination. Aspirate was negative for AFB and malignant cells. Direct KOH mount and GMS staining of the aspirate showed a few broad aseptate thin walled fungal hyphae with right angle branching, characteristic of Zygomycetes^1^ (Fig. 2). Fungal culture of the aspirate inoculated on to SDA media yielded white cottony colonies with no reverse pigmentation in 7 days. Lacto phenol cotton blue mount from the culture showed broad hyaline, thin walled aseptate fungal hyphae with right angle branching, typical of Zygomycetes fungus. He was started on multiple subcutaneous insulin regimens for glycemic control and intravenous Amphotericin-B (50mg/day) after which he showed improvement, clinically as well as radiologically. He is on regular follow up with no further complaints.

## DISCUSSION

Pulmonary Mucormycosis is less common opportunistic fungal disease, localized in the lungs or the mediastinum.^3^ Its estimated incidence was 1.7 cases per million people per year in the United States.^4^ In India, few cases have been reported but exact prevalence is not known. Mucormycosis is found in patients of wide age range but with a male predominance.^3^ It presents with fever, dyspnoea, cough and chest pain.^5^ Invasion of blood vessel by fungal hyphae, results in necrosis of tissue parenchyma, which may ultimately lead to cavitation and/or hemoptysis. Most common predisposing conditions for mucormycosis are uncontrolled diabetes mellitus, malignancy, chronic illnesses and transplants.^6^–^7^ Radiographically, a variety of findings may be present. In descending order of frequency these may include lobar consolidation, isolated masses, nodular disease, and cavitation.^8^ High-resolution chest CT scan is the most sensitive method of determining the extent of pulmonary mucormycosis. An important finding is expansion of the mass or consolidation across tissue planes, in particular, towards the great vessels in the mediastinum^9^ as is evident in this case. The most common method used for diagnosis is microscopic examination of specimens obtained via flexible fiber-optic bronchoscopy^3^. Due to refusal of the patient for bronchoscopy we opted for trans-thoracic route for collecting samples to reach the diagnosis in the present case. Though, usefulness of FNAC in lung lesions is well documented but for the diagnosis of pulmonary mucormycosis it is sparingly reported.^10^ Brochoalveolar lavage (BAL), a relatively safe diagnostic tool, may also allow the diagnosis of mucormycosis in cases where lung biopsy is contraindicated.^11^

Treatment of pulmonary mucormycosis is based on rapidity of diagnosis, reversal of the underlying predisposing factors (if possible) alongwith prompt institution of antifungal therapy and extensive surgical debridement.^5^ Pulmonary mucormycosis is relatively uncommon disease but with an increasing prevalence of diabetes in India, it is likely to be seen more commonly then before. A high level of clinical suspicion is important in any patient, in the presence of appropriate clinical setting and a non resolving pulmonary opacity despite antibiotic therapy. The diagnosis is generally missed in patients with diabetes mellitus, where tuberculosis is more common than any other cause of non resolving pneumonia. A high index of clinical suspicion should be maintained for pulmonary mucormycosis while investigating such patients for non resolving pneumonia.

In conclusion, pulmonary mucormycosis should be suspected in high risk patients for fungal pulmonary infections, particularly when they present with cavitation on chest radiograph with negative sputum smear for AFB in a country like India where prevalence of tuberculosis is very high. The diagnosis of fungal pneumonia requires the demonstration of fungi within the pulmonary parenchyma on lung biopsy; however, BAL, a relatively safe diagnostic tool, may also allow the diagnosis of mucormycosis in cases where lung biopsy is contraindicated.

## References

[1] Ribes JA, Vanover-Sams CL, Baker DJ (2000). Zygomycetes in human disease. Clin Microbiol Rev.

[2] Spellberg B, Edwards J, Ibrahim A (2005). Novel Perspectives on Mucormycosis: Pathophysiology, Presentation, and Management. Clin Microbiol Rev.

[3] Lee FY, Mossad SB, Adal KA (1999). Pulmonary mucormycosis: the last 30 years. Arch Intern Med.

[4] Rees JR, RW Pinner, RA Hajjeh, ME Brandt, Reingold AL (1998). The epidemiological features of invasive mycotic infections in the San Francisco Bay area, 1992–1993: results of population based laboratory active surveillance. Clin Infect. Dis.

[5] Tedder M, JA Spratt, MP Anstadt, SS Hegde, SD Tedder, Lowe JE (1994). Pulmonary mucormycosis: results of medical and surgical therapy. Ann. Thorac. Surg.

[6] Marr KA, RA Carter, Crippa F, Wald A, Corey L (2002). Epidemiology and outcome of mould infections in hematopoietic stem cell transplant recipients. Clin. Infect. Dis.

[7] Latif (1997). Pulmonary mucormycosis in persons on chelation therapy. Am J. Kidney Dis.

[8] McAdams HP, MRosado de Christenson, Strollo DC, Patz EF (1997). Pulmonary mucormycosis: radiologic findings in 32 cases. Am. J. Roentgenol.

[9] Reid VJ, Solnik DL, Daskalakis T, Sheka KP (2004). Management of bronchovascular mucormycosis in a diabetic: a surgical success. Ann. Thorac. Surg.

[10] Bakshi NA, Volke EE (2001). Pulmonary mucormycosis diagnosed by fine needle aspiration cytology. A case report. J Acta Cytol.

[11] Glazer M, Nusair S, Breuer R, Lafair J, Sherman Y, Berkman N (2000). The Role of BAL in the diagnosis of pulmonary mucormycosis. Chest.

